# CXCL10 Decreases GP73 Expression in Hepatoma Cells at the Early Stage of Hepatitis C Virus (HCV) Infection

**DOI:** 10.3390/ijms141224230

**Published:** 2013-12-13

**Authors:** Yuan Liu, Ziying Zou, Bing Zhu, Zonghai Hu, Ping Zeng

**Affiliations:** 1 Department of Microbiology and Immunology, Center of Laboratory Medicine, General Hospital of Chengdu Military Region of PLA, Chengdu 610083, China; E-Mails: zouziying49@163.com (Z.Z.); profzhubing@163.com (B.Z.); 13658027531@163.com (Z.H.); 2 Department of Clinical Chemistry, Center of Laboratory Medicine, General Hospital of Chengdu Military Region of PLA, Chengdu 610083, China; E-Mail: 18080933739@163.com

**Keywords:** hepatitis C virus, HCC, GP73, CXCL10, liver disease

## Abstract

Golgi protein 73 (GP73), which is up-regulated in hepatocellular carcinoma (HCC), has recently been identified as a novel serum marker for HCC diagnosis. Several reports also noted the increased levels of GP73 expression in chronic liver disease in patients with acute hepatitis of various etiologies, chronic Hepatitis C virus (HCV) infection and alcoholic liver disease. The molecular mechanisms of GP73 expression in HCV related liver disease still need to be determined. In this study, we aimed to evaluate the effect of HCV infection on GP73 expression. GP73 was highly expressed in Huh7, Hep3B, 293T and HUVEC cells, and was low-expressed in HepG2 cells. HCV infection led to down-regulation of GP73 in Huh7 and HepG2/CD81 cells at the early stage of infection. CXCL10 decreased GP73 expression in Huh7 and HepG2 cells. Up-regulation of GP73 was noted in hepatocytes with cytopathic effect at advanced stage of HCV infection, and further research is needed to determine the unknown factors affecting GP73 expression. In conclusion, our study provided additional evidence for the roles of GP73 in liver disease.

## 1. Introduction

Golgi protein 73 (GP73, also named Golgi Phosphoprotein 2 (GOLPH2) and Golgi membrane protein 1 (GOLM1)) is a type II Golgi membrane protein which has an *N*-terminal transmembrane domain and a *C*-terminal coiled-coil domain located on the lumenal surface of the Golgi apparatus [1]. Changes in expression levels of GP73 involves in pathogenesis of many diseases. Overexpression of GP73 is first reported in adult giant-cell hepatitis (GCH) [2] and is induced in hepatoma cells after adenovirus infection *in vitro* [3]. Recent studies have identified GP73 as a potential novel serum marker of cancer, including hepatocellular carcinomas (HCC) [4,5], lung cancer [6] and prostate cancer [7], and as an independent prognostic factor for tumor recurrence and poor overall survival [8]. Inconsistent result indicates that increased GP73 expression in HCC tissue does not correlate with survival [9]. On the contrary, the mRNA and protein level of GP73 is found down-regulated in gastric tumorous tissues [10]. In addition multiple single-nucleotide polymorphisms (SNPs) in GP73 are associated with the pathogenesis of Alzheimer’s disease (AD) [11,12].

Despite the large number of studies suggesting GP73 as a serum marker for HCC, at the present time, the absolute positive markers for HCC are still lacking, and even those characterized by high sensitivity and specificity (such as α-Fetoprotein (AFP), Glypican-3 (GPC3), Des-γ-Carboxy Prothrombin (DCP) and GP73, used alone or in combination) do not widely applied for clinical use. It is well known that the main established risk factors for HCC development are chronic viral hepatitis B and C infection. Liver cirrhosis may also lead to HCC development. HCV causes HCC probably via an indirect pathway by causing chronic inflammation and cirrhosis. Alcoholic liver disease and autoimmune liver disease are also associated with the development of HCC [13]. Interestingly, GP73 serum level is elevated in diverse liver diseases, including chronic hepatitis (caused by HBV or HCV infection), while the involved mechanisms is not well defined [3,14,15]. Thus, a better understanding of the molecular pathways of GP73 in HCV infection could potentially provide more information. Therefore, further research is desired to uncover the role of GP73 in HCV related liver disease and in which way GP73 is regulated by HCV infection. In this study we clarify that GP73 is down-regulated in hepatoma cells at the early stage of HCV infection, and CXCL10 plays an important role in this process. Moreover, GP73 is up-regulated in hepatocytes with CPE at the advanced stage of viral infection, and the factors involved in this situation need further research.

## 2. Results

### 2.1. GP73 mRNA Level Is Down-Regulated in HCV Infected Cells

**Figure 1.**
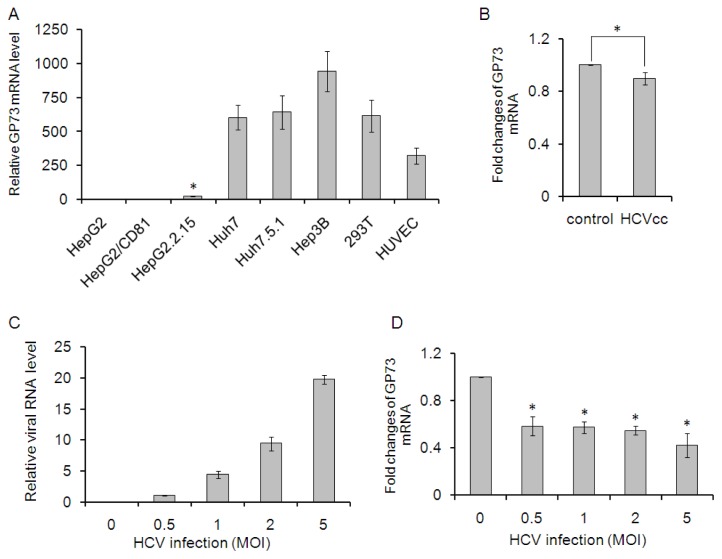
Effect of Hepatitis C virus (HCV) on Golgi protein 73 (GP73) expression in hepatocytes. (**A**) GP73 mRNA level in HepG2 cells was set as control. RT-PCR analysis was performed to detect GP73 levels in different cell lines compared to that in HepG2 cells; (**B**) RT-PCR analysis of GP73 mRNA levels in Huh7 cells at 24 h after HCV infection (* *p* < 0.05); (**C**) HepG2/CD81 cells were infected with the indicated concentrations of HCV for 24 h; HCV RNA levels (**C**) and GP73 mRNA levels (**D**) were quantified by real-time RT-PCR analyses. Experiments were performed three times with similar results. Graphs represent means ± SD, *n* = 3. (* *p* < 0.05, compared with control).

Kladney *et al*. reported that GP73 was present at high levels in HepG2.2.15 cells (a cell line that supports active HBV replication), but was absent in HepG2T14.1 cells (an HBV-transfected cell line that does not support HBV replication) and in HBV-free HepG2 cells [14]. To further clarify the expression of GP73 in different cell lines, the mRNA levels of GP73 in a serial of cells compared to that in HepG2 cells were detected through real time PCR. As shown in Figure 1A, HepG2/CD81 had a comparable level of GP73 to HepG2 cells. The mRNA level of GP73 in HepG2.2.15 was about 25-fold than that in HepG2 cells (*p* < 0.05). Huh7, Huh7.5.1, Hep3B, 293T and HUVEC cells showed dramatically higher levels of GP73, approximately 300 to 900 folds than that in HepG2 cells. To identify the effects of HCV infection on GP73 expression, GP73 mRNA level was detected in Huh7 cells 24 h after HCV infection. The mRNA levels of GP73 in HCV infected Huh7 cells were slightly down regulated (Figure 1B). HepG2 cells have been previously shown to support HCV cell entry if the missing receptor, CD81, is overexpressed [16]. Here, we labelled the HepG2 cells with overexpressed CD81 as HepG2/CD81. Similar results, that GP73 was down-regulated by HCV infection, were observed in HepG2/CD81 cells. The mRNA level of GP73 was reduced by about 50% in HepG2/CD81 cells with different doses of HCV infection. These results indicate that GP73 mRNA levels, either in cells with low level (HepG2/CD81) or high level (Huh7) of GP73, are down-regulated at the early stage of HCV infection.

### 2.2. CXCL10 Is Involved in the Down-Regulation of GP73

**Figure 2.**
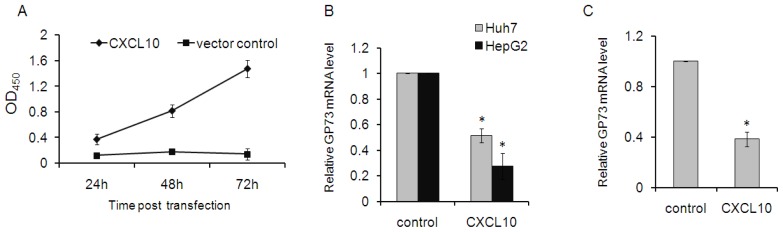
CXCL10 decreased GP73 expression. (**A**) HEK 293T cells were transfected with pcDNA-s-CXCL10 or control vector. CXCL10 in cell cultures were detected via ELISA at various time points after transfection; (**B**) Huh7 and HepG2 cells were treated with CXCL10 for 24 h, respectively. GP73 was measured by real-time RT-PCR; and (**C**) HEK 293T cells were transfected with pcDNA-s-CXCL10 or control vector. Cells were harvested at 72 h post-transfection. Relative levels of GP73 mRNA were measured by real-time RT-PCR. Experiments were repeated three times with similar results. Data represent means ± SD, *n* = 3. (* *p* < 0.05, compared with control).

HCV infection is characterized histologically by a persistent inflammatory response. The chemokine CXCL10 (also known as interferon-γ induced protein 10 or IP-10) plays a pivotal role in recruiting lympholeukocytes and macrophages to the liver [17]. Therefore, to investigate whether GP73 is regulated by this chemokine, recombinant CXCL10 was successfully produced in 293T cells. CXCL10 in supernatant of 293T cells reached a high level at 72 h after transfection (Figure 2A). Supernatant of 293T cells, transfected with either pcDNA-s-CXCL10 or mock vector, were harvested at 72 h after transfection and sterilized. Exactly 150 μL of the supernatant containing recombinant CXCL10 or control were added onto HepG2 and Huh7 cells and incubated for 2 days. GP73 levels were then measured in these cells. Stimulation with CXCL10 significantly decreased GP73 mRNA levels both in Huh7 and HepG2 cells (Figure 2B). Interestingly, GP73 mRNA levels were also down-regulated in 293T cells transfected with CXCL10 plasmids (Figure 2C). This down-regulation of GP73 was also observed in Huh7 cells transfected with plasmids of CXCL10 (data not shown).

### 2.3. GP73 Is Up-Regulated in Cells with Cytopathic Effect Induced by HCV Infection

**Figure 3.**
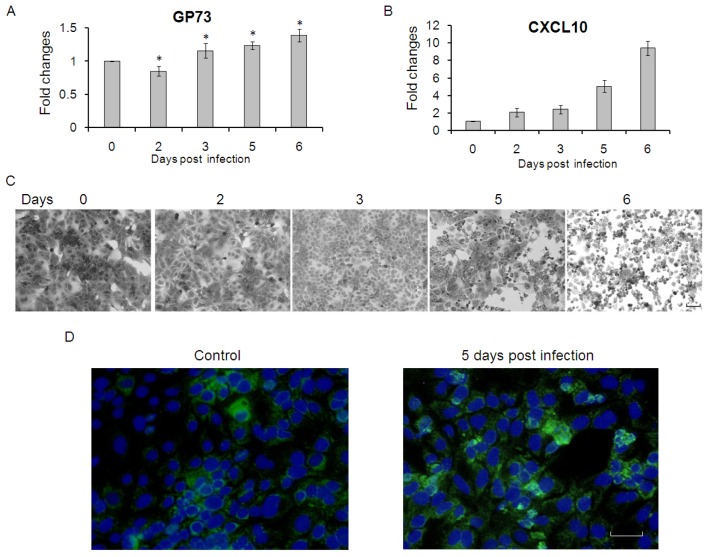
GP73 is up-regulated in Huh7.5.1 cells with cytopathic effect. (**A**) GP73 and CXCL10 (**B**) were measured by real-time RT-PCR in Huh7.5.1 cells at 0, 2, 3, 5 and 6 days after HCV infection (*MOI* = 10), (* *p* < 0.05, compared with control); (**C**) Crystal violet (CV) staining of Huh7.5.1 cells at 0, 2, 3, 5 and 6 days after HCV infection (*MOI* = 10). (Scale bar = 50 μM); and (**D**) Detection of GP73 protein by immunofluorescence assay. (Green for GP73; Blue for nuclei, Scale bar = 50 μM).

Significant increases of GP73 were previously found in liver disease due to viral causes (HBV, HCV) or nonviral causes (alcohol-induced liver disease, autoimmune hepatitis), while the sera or liver tissue specimen were mainly from chronic liver disease. Therefore, to further observe the long-term effects of HCV infection on GP73 expression in hepatoma carcinoma cells, we detected the GP73 mRNA level in Huh7.5.1 cells at different time points after HCV infection. As shown in Figure 3A, GP73 was down-regulated at 2 days post infection, while up-regulated after 3 days. CXCL10, which decreased the expression of GP73, were also up-regulated at each time points after HCV infection (Figure 3B). It was worth to note that Huh7.5.1 cells showed marked cytopathic effect (CPE) at later days after HCV infection (Figure 3C), which probably caused the increased expression of GP73. The levels of GP73 protein were up-regulated at later days of HCV infection (Figure 3D).

**Figure 4.**
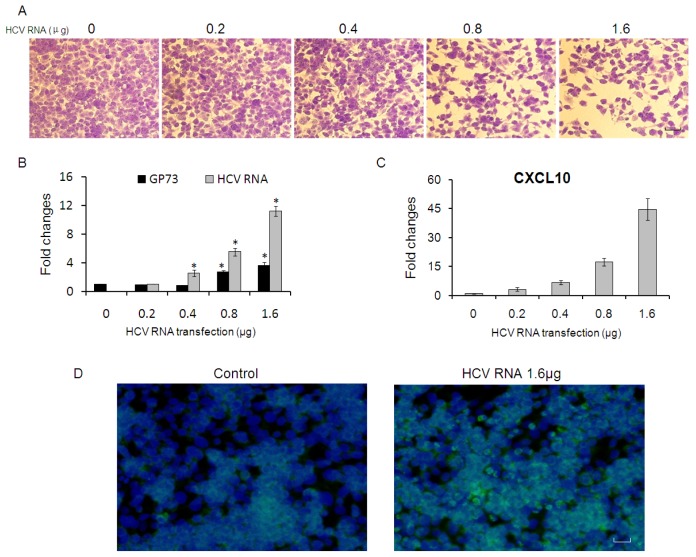
HepG2 cells were transfected with 0, 0.2, 0.4, 0.8 and 1.6 μg of HCV RNA. (**A**) CV staining was performed at 48 h post transfection. (Scale bar = 50 μM); GP73 and HCV RNA levels (**B**) and CXCL10 (**C**) in HepG2 cells were detected by real-time RT-PCR. Data were shown the means and standard errors of three replicate assays; (* *p* < 0.05, compared with control); and (**D**) Detection of GP73 protein by immunofluorescence assay. (Green for GP73; Blue for nuclei; Scale bar = 50 μM).

Similar results were also found in HepG2 cells. Marked CPE were shown in HepG2 cells transfected with more than 0.8 μg of HCV RNA (Figure 4A), and GP73 mRNA levels in these CPE cells were greatly up-regulated (Figure 4B). CXCL10 were up-regulated in HepG2 cells transfected with each dose of HCV RNA (Figure 4C). The levels of GP73 protein were shown in Figure 4D and increased GP73 were detected in HepG2 cells transfected with 1.6 μg of HCV RNA.

**Figure 5.**
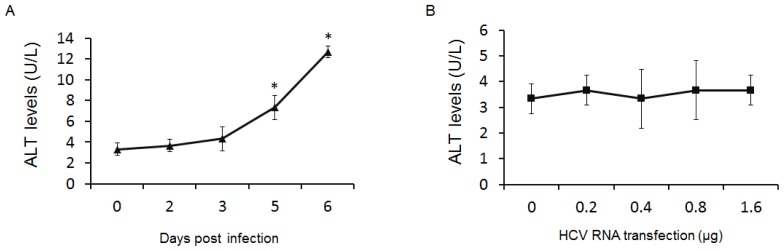
ALT levels in cell culture supernatant. (**A**) Huh7.5.1 cells were infected with HCV. Cell culture supernatants were collected for ALT detection at different time points after infection (* *p* < 0.05, compared with 0 day); and (**B**) HepG2 cells were tranfected by different dose of HCV RNA. ALT levels in supernatant were detected at 24 h post tranfection.

Increased levels of ALT were found in the supernatant of Huh7.5.1 cells at later days of HCV infection (Figure 5A). However, no significant changes of ALT were detected in that of HepG2 cells after 24 h of transfection (Figure 5B).

### 2.4. GP73 Had No Impact on Proliferation of Vascular Endothelial Cells

**Figure 6.**
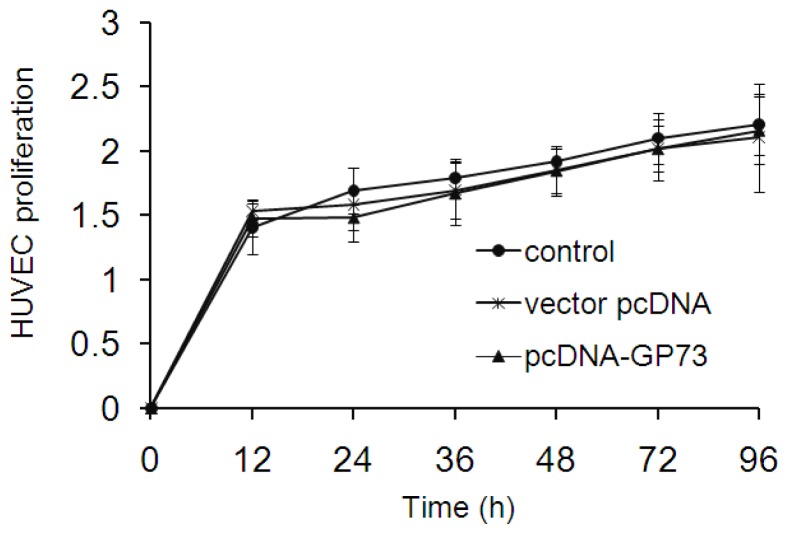
Effects of GP73 on the proliferation of vascular endothelial cells. Human umbilical vein endothelial cells (HUVECs) stably expressing GP73 were obtained as described in the methods section. Percentage of viability was obtained by comparing values to that of HUVECs in a control group. HUVECs proliferation variation was obtained by calculating the changes of HUVECs to baseline. Data was generated from HUVECs with three replicates and means ± SD values were shown.

Hepatocellular carcinoma (HCC) is characterized by highly vascularized and rapid tumor progression which has been considered as the main reason for its devastating outcome. Proliferation of vascular endothelial cells plays an important role in tumor growth. Wei *et al.* found that GP73 may clearly prompt the proliferation of LX2 cells (hepatic stellate cell line), but without any effect on HepG2 cells *in vitro* [18]. Similar results, that overexpression of GP73 had no impact on HepG2 cell proliferation, were observed in our study (data not shown). To further detect if GP73 plays a role in the proliferation of vascular endothelial cells, we detected the cell proliferation in HUVEC with or without overexpression of GP73. The results showed that overexpression of GP73 had limited impact on proliferation of HUVEC cells (Figure 6).

## 3. Discussion

More and more studies have revealed that the level of serum GP73 has great value for diagnosis of hepatocellular carcinoma and several other cancers. The mechanisms responsible for this variability are still unknown. Several investigations have demonstrated that GP73 protein is overexpressed in a variety of acute and chronic liver diseases and its concentration was significantly correlated with the grading of liver fibrosis [14,15]. However, the relationship between serum GP73 concentration and staging or grading of chronic liver disease is still a problem to be solved. Furthermore, the utility of this biomarker remains limited, which is largely due to the lack of quantitative evidences. In this study, we provided additional information for the association between GP73 regulation and liver disease.

We found that GP73 was down-regulated in hepatocytes early after HCV infection. HCV infection is associated with a complex dysregulation of the cytokine/chemokine network. Patients with chronic HCV infection are at risk for developing cirrhosis and HCC [13]. Several factors influence this process, including proinflammatory cytokines such as CXCL10 which was up-regulated in HCV infected hepatocytes. GP73 levels in hepatoma carcinoma cells (Huh7 and HepG2) stimulated with CXCL10 were decreased. Similar results were observed in Huh7 and human embryonic kidney (293T) cells transfected with CXCL10 plasmids. It is still not known how CXCL10 mediates the down-regulation of GP73. CXCL10 elicits its effects by binding to the cell surface chemokine receptor CXCR3. Therefore the effects of other two CXCR3 ligands (CXCL9 (Mig) and CXCL11 (ITAC)) on GP73 expression need further research. Interestingly, GP73 levels were significantly up-regulated in hepatocytes with marked CPE induced by boost HCV RNA transfection in HepG2 cells or in advanced stage of HCV infected Huh7.5.1 cells, regardless of the dramatically increased CXCL10 levels in these cells. The ALT levels increased greatly in the supernatant of HCV infected Huh7.5.1 cells at later days. Wei *et al.* [18] have shown that serum GP73 concentration was correlated with ALT in patients with ALT > 80 U/L, but nearly normal ALT was not. GP73 were also correlated with total bilirubin, which together indicates that GP73 expression is correlated with liver injury.

In normal livers, GP73 is considered to presenting in biliary epithelial cells, whereas hepatocytes show little or no expression of GP73 [2]. Overexpressed GP73 was found in hepatocytes from patients with acute hepatitis, autoimmune hepatitis, chronic HCV infection and alcoholic liver disease. Up-regulation of GP73 is also induced in human Hep3B hepatoma cells infected with human adenovirus, and the *C*-terminus of E1A is required for GP73 expression [3]. Studies also found that IFN-γ and TNF-α were able to increase and decrease GP73 expression in SK-Hep-1 cells [14]. Levels of GP73 mRNA and protein were up-regulated in HepG2 cells following treatment with either proinflammatory cytokine IL-6 or the related cytokine oncostatin M (OSM) [19]. Combined with the findings in our study, CXCL10 is involved in the down-regulation of GP73 at early stage of HCV infection, while the factors that increased the expression of GP73 at advanced stage of liver disease are still not well defined and further research are urgently needed to clarify these factors.

Since the high GP73 serum concentration was observed in patients with advanced liver disease and some tumors, which suggested that soluble GP73 may be playing a role in disease progression. Previous study has found that GP73 may obviously prompt proliferation of LX2 cells [18]. We further characterized that overexpression of GP73 had limit effect on the proliferation of vascular endothelial cells. The role of GP73 in disease progression is still an important problem to be solved.

In conclusion, GP73 is an interesting molecule that deserves further investigation. The molecular mechanism of up-regulation of GP73 in advanced liver disease still needs to be addressed. Moreover, researchers pointed out that increased expression of GP73 in hepatocytes appears to be a general feature of advanced liver disease, and may be regulated via distinct pathways that involve hepatotropic viruses or cytokines. Further studies are warranted to be able to assess the role of GP73 in the progression of liver disease.

## 4. Materials and Methods

### 4.1. Cells and Plasmids Construction

HEK 293T, HepG2, HepG2.2.15, Hep3B, Huh7, Huh7.5.1 and human umbilical vein endothelial cells (HUVEC) were grown under standard conditions in Dulbecco’s modified Eagle’s medium supplemented with 10% fetal bovine serum, 2 mM l-glutamine, 0.1 mmol/L nonessential amino acids (NEAA) and 1% penicillin-streptomycin (Gibco BRL, Grand Island, NY, USA). HepG2/CD81 was generated by overexpression of human CD81 in HepG2 cells [20].

Encoding genes of *GP73* (GI: 7271866) and *CXCL10* (GI: 323422857) were amplified from Huh7 cell cDNA by polymerase chain reaction (PCR). The forward and reverse primers used were shown in Table 1, and the restriction sites for Hind III (upstream) and BamH I (downstream) are shown as italic and underline letters. PCR-based addition of secretory signal was performed for extra-cellular protein secretion at 5′ end of CXCL10 cDNA construct [21]. All PCR products were digested with Hind III and BamH I (Takara Co, Otsu, Japan) and used for ligation into plasmid pcDNA3.1, which was named as pcDNA-GP73 and pcDNA-s-CXCL10 respectively.

Purified RNA or plasmids was transfected into HepG2, HUVEC or Huh7 cells using lipofectamin 2000 (Invitrogen, Carlsbad, CA, USA) according to the manufacturer’s instructions.

### 4.2. Viral RNA *in Vitro* Transcriptions

The plasmid pFL-J6JFH1 (a gift from Charles Rice, Rockefeller University, New York, NY, USA) was first linearized by XbaI (Takara Co, Otsu, Japan). The mixture was precipitated by 0.1 volumes of NaAc (3M pH = 5.2) and 2 volumes of Ethanol. The precipitation was resolved with diethyl pyrocarbonate (DEPC)-water, and used as templates to generate virus genome RNA using *in vitro* transcription kit (Ambion, Austin, TX, USA) according to the manufacturer’s recommendations. Then these RNA were purified with tris-saturated phenol: chloroform: isoamyl alcohol (25:24:1). Before transfection 1.5% agarose gel electrophoresis was performed to check the integrity and purity of viral genome RNA.

Cell-culture-derived HCV (HCVcc) were produced by transfection of Huh7 cells with HCV genome RNA as described [22]. The supernatant were harvested at 72 h after transfection and purified through a 0.45 μM filtration membrane before use.

### 4.3. Real Time PCR

For GP73 gene expression analysis, total RNA was isolated from cells using Trizol agent (Invitrogen, Carlsbad, CA, USA) and reverse transcribed to cDNA using the cDNA synthesis kit (Invitrogen, Carlsbad, CA, USA). Each cDNA sample was analyzed using the StepOnePlus real-time PCR system (Applied Biosystems, Foster city, CA, USA) using SYBR Premix ExTaq2 (Takara Co, Otsu, Japan) according to the manufacturer’s protocol. The primers used for gene expression analysis were shown in Table 2. GAPDH was used as an internal control.

### 4.4. Cytokine Stimulation

CXCL10 were produced as described [21,23]. Briefly 293T were transfected with pcDNA-s-CXCL10 or mock vector control. Cell culture were harvested at different time points post transfection and sterilized through a 0.45 μM filter cartridge. The secretion levels of CXCL10 in cell culture were assayed via Human CXCL10/IP-10 Quantikine ELISA Kit (R & D Systems, Minneapolis, MN, USA) according to the producer’s protocol. Huh7 and HepG2 cells were seeded onto 24-well plates (2 × 10^4^ cells/well). Twenty-four hours later, culture medium was removed and replaced with 500 μL fresh medium containing 150 μL of culture from 293T cells transfected with pcDNA-s-CXCL10 or mock vector control. Cells were incubated for 24 h and total RNA was isolated for RT-PCR analysis.

### 4.5. Immunofluorescence Assay

Immunofluorescence assay was performed as described [14]. Briefly, cultured cells were fixed for 20 min in ice-cold methanol, which were then washed with PBS. Cells were incubated for 2 h with a blocking solution containing 5% nonfat dry milk in PBS, which was followed by 1-hour incubation with polyclonal rabbit GP73 antibody (1:500 in blocking solution) (TIYO Biotechnology Corporation, Shanghai, China) at room temperature. Then cells were washed again and incubated with goat anti-rabbit IgG (1:400 *v*/*v*) conjugated to Alexa Fluor 488 (Invitrogen, Carlsbad, CA, USA). At last, cells were stained with 4,6-diamidino-2-phenylindole (DAPI, 1:10000) and viewed using Immunofluorescence microscope (Olympus Corporation, Tokyo, Japan).

### 4.6. Cell Proliferation Assay

Cultured HUVEC cells were divided into three groups: transfected with recombinant pcDNA-GP73, transfected with pcDNA3.1 mock vector and untransfected cells. Cells were cultured in DMEM medium containing 10% fetal bovine serum and 350 mg/L G418 (Gibco BRL, Grand Island, NY, USA). Resistant clones could be detected two weeks later and were selected to further expand.

Cell proliferation was monitored using Cell Counting Kit (CCK8) (Dojindo, Japan). HUVEC cells with overexpressing GP73 or control were allowed to grow in 96-well plates (3000 cells/well). Cell proliferation was measured at 12 or 24 h intervals following the manufacturer’s protocol. All experiments were performed in triplicates.

### 4.7. Statistical Analysis

Data are presented as means ± standard error (SD). Statistical significance of differences was examined by one-way ANOVA and Dunnett’s test. All data were analyzed using the SPSS 18.0 computer program (IBM, New York, NY, USA, 2009), and significant difference was defined as *p* < 0.05.

## 5. Conclusions

In the present study, we identified that GP73 is down-regulated at the early stage of HCV infection while is up-regulated at advanced stage of HCV infection *in vitro*. The chemokine CXCL10 was thought to induce decreased expression of GP73. CXCL10 stimulation leads to down-regulation of GP73 in Huh7 and HepG2 cells. Overexpression of GP73 in vascular endothelial cells had no impact on cell proliferation.

## Figures and Tables

**Table 1.** Primer sequences for plasmids construction.

Gene	Primer sequences (5′-3′)
*GP73*	Forward:CATAG*AAGCTT*CTCCAGACACGGATCATGGAGCTGGAAGGC
	Reverse:GAGAG*GGATCC*TCAGAGTGTATGATTCCGCTTTTCACGCTG
*CXCL10*	Forward:CATAG*AAGCTT*CTCGAGATGCTCCTGGCTGTTTTGTACTGCCTGCTGTGGAGTTTCCAGACCTCCGCTGGCCATTTCCCTAGAATGAATCAAACTGCCATTCTG
	Reverse:GAGAG*GGATCC*AACATAGCACCTCAGTAGAGC

**Table 2.** Primer sequences for RT-PCR.

Genes	Forward primer (5′-3′)	Reverse primer (5′-3′)
*HCV*	CTTCACGCAGAAAGCGTCTA	CAAGCACCCTATCAGGCAGT
*GP73*	TTGGTAACAGCAAGTCCCAGACA	ACCACCTGGATCTCATTGGTTTC
*CXCL10*	AGGAACCTCCAGTCTCAGCA	CAAAATTGGCTTGCAGGAAT
*GAPDH*	TGGGCTACACTGAGCACCAG	AAGTGGTCGTTGAGGGCAAT

## Abbreviations

GP73: Golgi protein 73
HCC: Hepatocellular carcinoma
HCV: Hepatitis C virus
GOLPH2: Golgi Phosphoprotein 2
GOLM1: Golgi membrane protein 1
SNPs: Single-nucleotide polymorphisms
AD: Alzheimer’s disease
AFP: Alpha-Fetoprotein
GPC3: Glypican-3
DCP: Des-γ-Carboxy Prothrombin
CXCL10: C-X-C motif chemokine 10
CPE: Cytopathic effect
HUVEC: Human umbilical vein endothelial cells
IFN: Interferon
TNF: Tumor necrosis factor
IL-6: Interleukin-6
OSM: oncostatin M
